# Inadequate Dosage May Lead to the Recurrence of Postoperative Pulmonary Hypertension in Patients With Congenital Heart Disease

**DOI:** 10.3389/fphar.2021.660405

**Published:** 2021-04-29

**Authors:** Xinmei Li, Te Li

**Affiliations:** ^1^ Yunnan Provincial Key Laboratory of Pharmacology, Kunming Medical University, Kunming, China; ^2^ Department of Pharmacy, Fuwai Yunnan Cardiovascular Hospital, Kunming, China

**Keywords:** pulmonary artery hypertension, interventional closure, surgery, doses administered, sildenafil

## Abstract

**Background:** Pulmonary arterial hypertension (PAH) associated with congenital heart disease (PAH-CHD) occurs predominantly among patients with uncorrected congenital heart disease (CHD). It is an intractable problem to control PAH continuously and stably after an operation.

**Methods:** 1) OPLS-DA combined with S-plot was used to retrospectively analyze the results of preoperative and postoperative PAH and 39 biochemical indicators of 235 patients admitted to Fuwai Yunnan Cardiovascular Hospital from January 2019 to December 2020. 2) Combined with Meta-analysis, the recurrence in postoperative PAH was analyzed in terms of operation factors, doses administered, and age factors.

**Results:** 1) 4 indicators (PAH, RBC, HGB, and CO_2_) that reflect the prognosis of patients had been found by OPLS-DA combined with S-plot. 2) The recurrence rate of postoperative PAH was 37.02%. The comprehensive therapeutic effect of interventional closure was better than that of surgical operation. PAH was not significantly higher again in patients who received either the instruction dose or the literature dose. Postoperative combination therapy (bosentan and sildenafil) was more effective than bosentan alone. Recovery after treatment was better in infants than in the other four age groups.

**Conclusion:** OPLS-DA combined with S-plot was used for the first time to analyze clinical examination data. In this study, this method proved to be a feasible method for analyzing clinical data We recommend interventional closure as the first choice for patients with PAH-CHD. For postoperative oral therapy, we recommend the combination therapy (bosentan with sildenafil). To prevent the recurrence, the dose should be strictly prescribed according to the instructions, literature, or body surface area converted. Moreover, we recommend treatment at a young age in these patients.

## Introduction

Pulmonary arterial hypertension (PAH) associated with congenital heart disease (PAH-CHD) occurs predominantly among patients with uncorrected congenital heart disease (CHD). These patients have small cardiac defects (usually ventricular septal defects <1 cm and atrial septal defects <2 cm) (Nazzareno et al., 2016). A significant number of patients have persistent or recurrent PAH after congenital heart repair surgery or percutaneous closure (Xi et al., 2019). Some studies speculate that this phenomenon may be due to high surgical risk (Mclaughlin et al., 2009; Kiefer and Bashore, 2011; Melby et al., 2011). However, no studies had shown the cause of recurrence and no detailed analysis of the indicators that lead to PAH recurrence.

The orthogonal projections to latent structures discriminant analysis (OPLS-DA) is an extension of the principal component analysis (PCA) and currently used mainly for metabolomics data analysis (Zhao et al., 2017). In contrast to basal metabolomics studies, data collection in the clinic is limited, and patient data are only collected at the time of initial consultation and routine examinations. Doctors are accustomed to ignoring normal values in these examinations and interpret abnormal values based on anecdotal rather than scientific evidence. Studies had shown that PCA can save doctors’ time in analyzing clinical indicators (Li and Li, 2020). In this study, OPLS-DA was used for the first time to analyze the routine clinical examination to identify reliable indicators related to the prognosis of patients with PAH-CHD.

Currently, randomized controlled trials of bosentan and sildenafil have been conducted in children (More et al., 2016; Kelly et al., 2017). However, most studies have mostly reported short-term preoperative use of a single drug (bosentan or sildenafil), with no reports of long-term (>9 months) postoperative use (Gilbert et al., 2005; Ashraf and Midany, 2013). In the present long-term follow-up study, we retrospectively analyzed data from patients with PAH-CHD who underwent correction (interventional closure or surgery).

## Materials and Methods

### Patients’ Characteristics

The institutional review board approved this study and waived the requirement for individual consent because of its retrospective nature. From January 1, 2019 solstice to December 31, 2020, 235 patients with PAH-CHD were admitted and treated in Fuwai Yunnan Cardiovascular Hospital, Kunming, China. We collected the data (PAH, postoperative medications, and 39 other indications from routine examinations) on these patients who underwent correction. Clinical and baseline characteristics of the data were preliminarily analyzed.

### Orthogonal Projections to Latent Structures Discriminant Analysis and S-Plot

OPLS-DA of SIMCA 14.1 (Umetrics, Kinnelon, New Jersey) was utilized to analyze and compare the indicators of the preoperative and follow-up groups, and S-plot was made. Forty indicators were including PAH by echocardiography and routine examinations (liver function examination item, kidney function examination item, blood lipid examination item, blood electrolyte, blood cell examination item). Then, we identified the indicators in S-plot where the difference was more than 0.5 and less than −0.5. The pre- and post-operative values of these different indicators were compared. The PAH indicators of the patients were analyzed over 2 years, including the preoperative group, the postoperative group, and the follow-up group.

### Analysis of the Causes of Pulmonary Arterial Hypertension Changes

#### Literature Search and Inclusion Criterion

We conducted a comprehensive search for studies of patients with PAH-CHD who underwent correction. The literature was searched using MEDLINE, CNKI, WAN FANG, and Cochrane Library (up to December 2020). The screening methods of the three times are presented in Table 1.

**TABLE 1:** Inclusion and exclusion criterion for three times literature retrievals in Chinese and English.

Criterion number	Keywords	Inclusion	Exclusion
1	PAH-CHD, interventional closure or surgery, follow up	Postoperative pulmonary arterial systolic blood pressure was followed up	1) The study had no record of PAH; 2) Study combined with multiple drug therapy
2	PAH-CHD, sildenafil or bosentan	1) The dosage was clear; 2)The patient's weight and age were clear	1) The study had no record of PAH; 2) Study combined with multiple drug therapy
3	PAH-CHD, interventional closure or surgery, sildenafil or bosentan	The patient was given medication postoperatively	Patients were given medication during perioperative period

#### Interventional Closure and Surgery

We counted 235 cases of PAH recurrence between interventional closure and surgery. To further understand this difference, we performed the literature search by criterion 1. Firstly, the mean, sd, and n values of PAH in the study were formally adjusted and compared between preoperative, postoperative, and follow-up groups (Li and Li, 2020). Secondly, we performed a conventional meta-analysis to compare the therapeutic and prognostic outcomes of different operations. Differences were assessed by odds ratio (OR) with 95% confidence intervals (CIs). The possibility of publication bias was estimated using funnel plots. Heterogeneity among studies was evaluated by calculating *p*-value and the *I* ^2^ measure of inconsistency, which was considered significant if *p* < 0.10 and *I* ^2^ > 50%. All calculations were carried out using Review Manager 5.3 (The Nordic Cochrane Center, Copenhagen, Denmark).

#### Drug Factors

We reviewed the instructions for use of sildenafil and bosentan to ensure correct dosing. The literature was screened according to the criterion 2. At the same time, the dose of the two drugs was calculated according to the patient’s body surface area. The therapeutic and prognostic effects of three doses of sildenafil and bosentan were compared, including body surface dose (DBSA), literature dose (DL), and case review (DCR). Literature was screened according to the criterion 3 to compare the postoperative oral efficacy of the two drugs.

#### Age Factors

We divided 235 patients into five groups, namely infants (<1 year), toddlers (1–6 years), children (7–14 years), adolescents (15–17 years), and adults (≥18 years). Recurrence, preoperative PAH, and treatment outcomes were analyzed for each group.

#### Data Analysis

Statistical analysis was performed using *t*-test and ANOVA. The *t*-tests and graphs of each drug safety indicators were applied in GraphPad Prism 8 (GraphPad Software, San Diego, Canada). Results were considered statistically significant when the *p*-value was <0.05.

## Result

### Patients’ Characteristics

Among 235 patients, the number of juvenile patients was approximately two times that of adult patients, but the proportion of PAH recurrences in the juvenile group was half that of adult group (37.02%) (Table 2).

**TABLE 2:** Clinical and baseline characteristics of 235 patients.

Characteristic	Minor group (*n* = 161)	Adult group (*n* = 74)
Gender, male/female	69/92	29/45
Weight, kg	(19.83 ± 18.96)	(54.16 ± 12.79)
Age, year	(6.42 ± 5.38)	(41.81 ± 14.23)
Right atrial pressure, mm Hg	(63.42 ± 26.81)	(68.51 ± 14.53)
Pulmonary vascular resistance, wood	(18.41 ± 13.73)	(18.32 ± 27.58)
Pulmonary arterial systolic pressure, mm Hg	(73.74 ± 20.87)	(78.11 ± 26.56)
Mean pulmonary arterial, mm Hg	(55.46 ± 15.57)	(48.67 ± 22.23)
Interventional closure/surgery	43/118	20/54
PAH recurrence, %	29.19%	54.05%

### Orthogonal Projections to Latent Structures Discriminant Analysis and S-Plot

**FIGURE 1:**
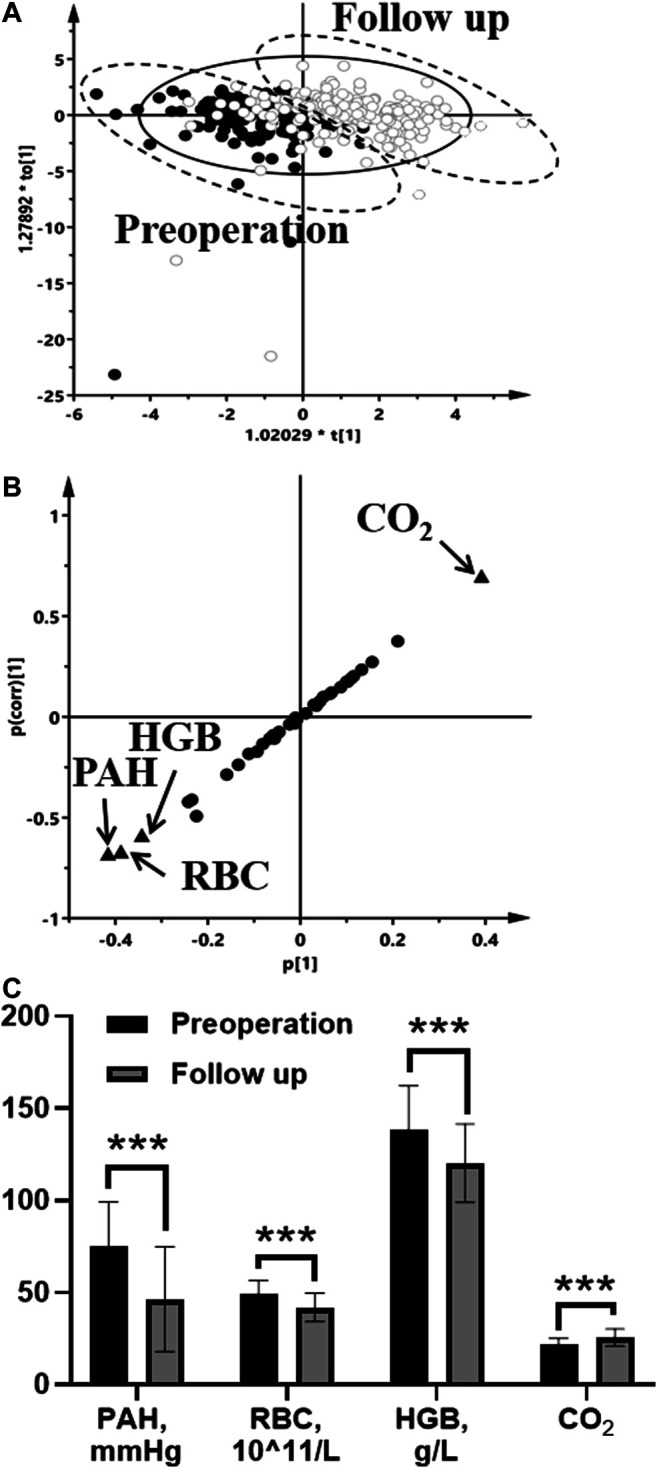
Indicators analysis in the preoperative group and the follow-up group by OPLS-DA score plots **(A)**, S-plot **(B)**, and comparison of four difference indicators **(C)** (●, the preoperative group in score plots; ○, the follow-up group in score plots; four differential indicators are labeled in S-plots). Statistical analysis was performed using *t* test. *p < 0.05; **p < 0.01; ***p < 0.001.

Using OPLS-DA with S-plot, we found four indicators: PAH, red blood cell (RBC), hemoglobin (HGB), and carbon dioxide levels in the blood (CO_2_) (Figure 1). By analyzing the data of 4 indicators, it was found that *t*-test result is consistent with the result of the S-plot results.

**FIGURE 2:**
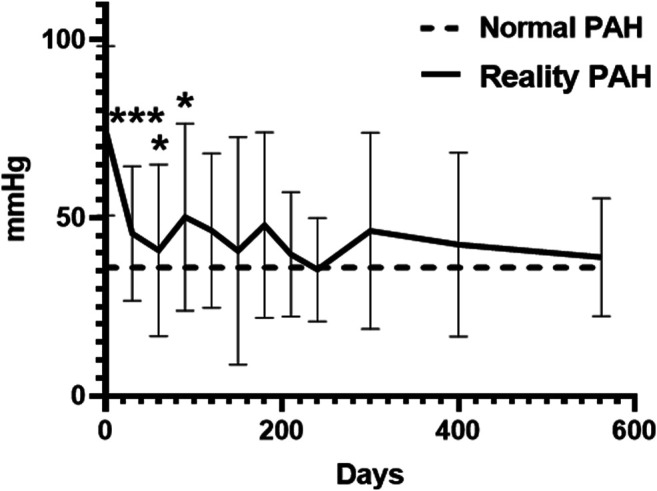
PAH changes in 2 years including preoperation, postoperation, and follow up.*p < 0.05; **p < 0.01; ***p < 0.001.

We performed a statistical analysis of PAH, including preoperative, postoperative, and follow-up periods (Figure 2). After treatment (interventional closure or surgery), PAH improved significantly, with a mean decrease of 28.85 mmHg. However, it increased in the postoperative follow-up group 60–90 days (*p* < 0.05). There were no subnormal values of pulmonary artery systolic pressure (36 mmHg) in the preoperative, postoperative, and follow-up groups, so we used PAH to represent pulmonary artery systolic pressure.

### Cause Analysis of Pulmonary Arterial Hypertension Changes

#### Interventional Closure and Surgery

Echocardiography of pre-treatment PAH was performed in 10 patients only and was not included in the analysis of PAH results. From a review of 224 cases, the recurrence rate of PAH of surgery (50.83%, *n* = 172) was higher than that of interventional closure (41.26%, *n* = 52). We selected nine Chinese literature about patients with PAH-CHD who underwent correction according to criterion 1 (Table 3) (Zhang et al., 2012; Jing, 2013; Zhao, 2013; Cao et al., 2016; Jing et al., 2016; Sun et al., 2016; Zhang et al., 2016; Chao and Zhao, 2017; Wang et al., 2020). In Table 3, we also recorded the PAH of both groups in this case review.

**TABLE 3:** PAH in nine studies including preoperative group, postoperative group, and follow-up group.

Study	Treatment	Pre-operation PAH			Post-operation PAH			Follow-up PAH		
		Mean	SD	N	Mean	SD	N	Mean	SD	N
Cao2017	Interventional closure	62.60	9.90	23	46.00	7.20	23	35.20	4.60	23
Cao2016	Interventional closure	46.90	8.49	25	35.66	10.53	25	NA	NA	NA
Jing2013	Interventional closure	59.24	15.00	62	NA	NA	NA	25.77	6.50	62
Sun2016	Interventional closure	30.30	6.30	28	27.30	5.60	28	24.80	2.10	28
Wang2020	Interventional closure	95.18	24.14	34	53.41	16.37	34	40.33	12.64	34
Zhang2012	Interventional closure	76.00	51.00	43	46.26	17.26	43	NA	NA	NA
Zhang2018	Interventional closure	58.00	24.00	64	36.00	11.00	64	NA	NA	NA
Zhao2013	Surgical operation	69.40	24.50	60	32.60	8.20	60	NA	NA	NA
Jing 2016a	Surgical operation	65.30	9.50	6	24.00	1.40	6	32.10	3.50	6
Jing 2016b	Surgical operation	72.20	11.40	14	33.20	4.20	14	43.80	4.30	14
Jing 2016c	Surgical operation	83.50	13.60	12	44.80	4.50	12	53.50	5.40	12
Case review (closure)	Interventional closure	79.94	30.24	52	60.63	32.43	52	54.12	26.98	52
Case review (operation)	Surgical operation	70.77	22.64	172	39.76	18.04	172	44.25	22.37	172

NA means there is no relevant value in the references.

**FIGURE 3:**
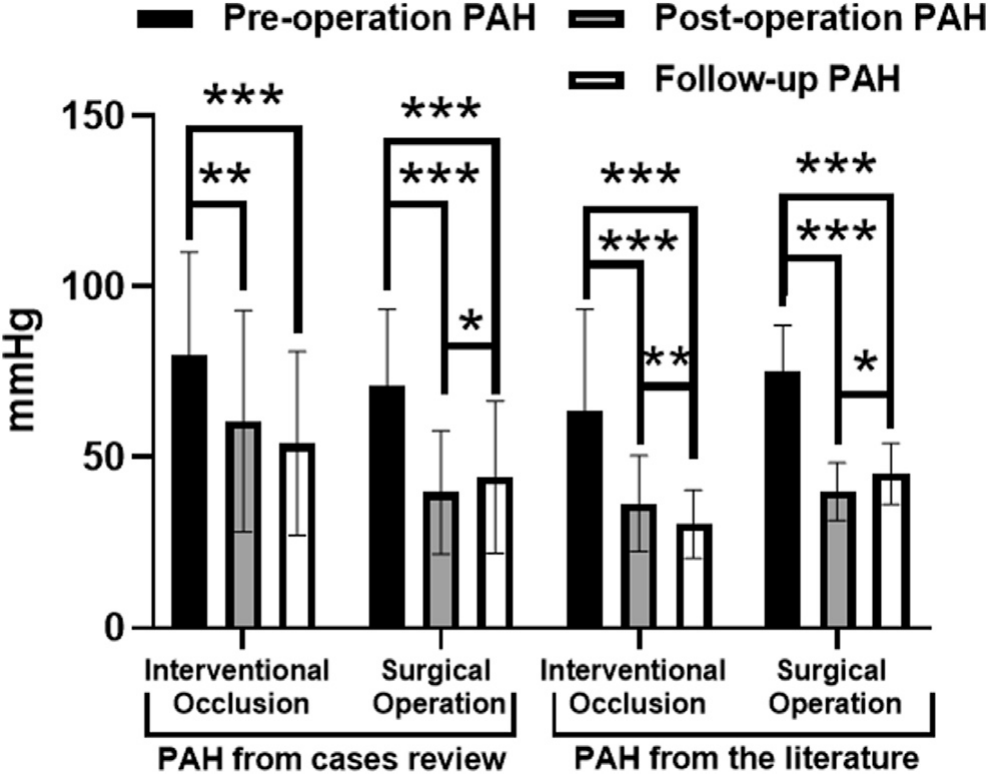
PAH in case review and nine studies between interventional occlusion and surgical operation including preoperation, postoperation, and follow up.*p < 0.05; **p < 0.01; ***p < 0.001.

**FIGURE 4:**
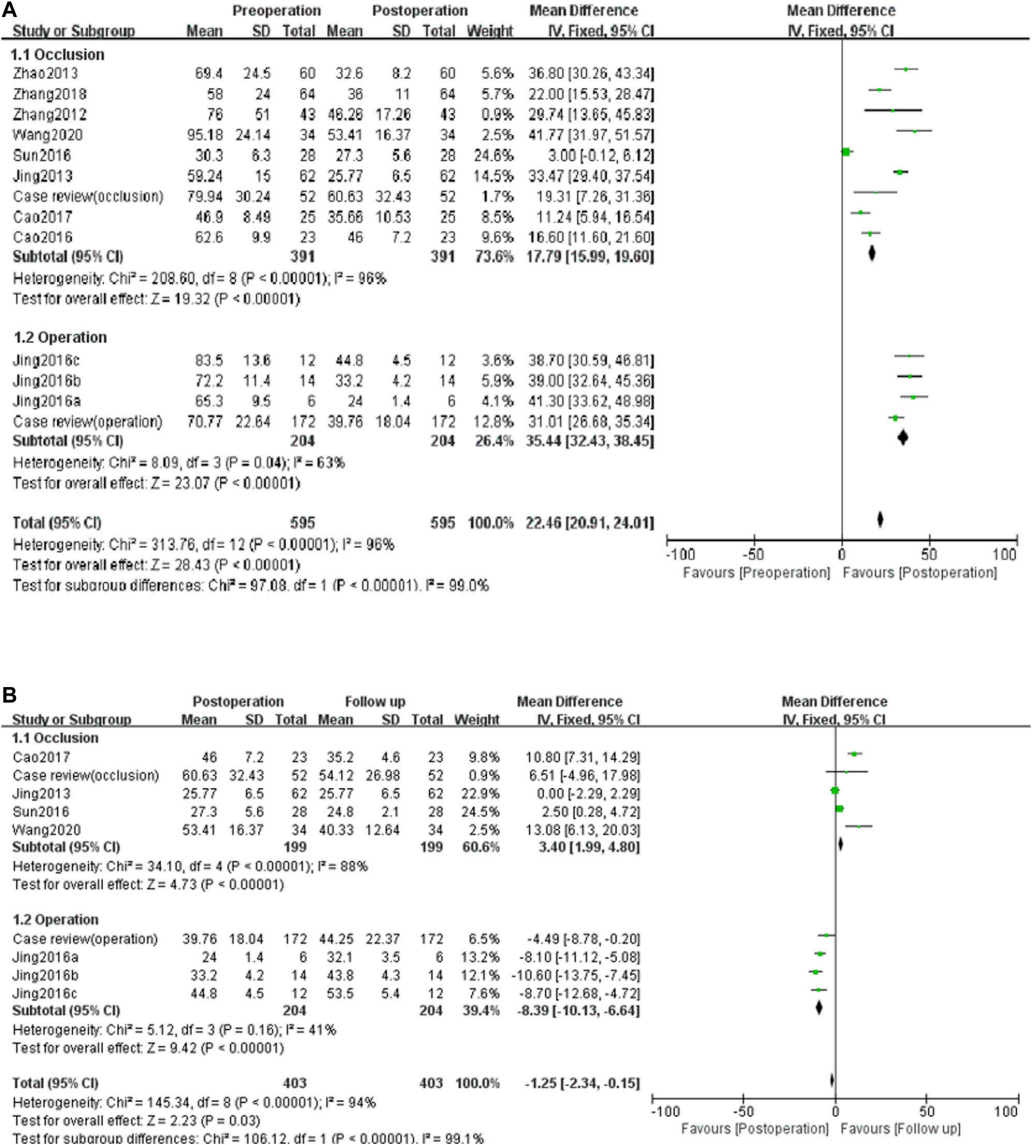
Meta-analysis to compare the therapeutic effects **(A)** and prognostic effects **(B)** of different operation.

As shown in Figure 3, similar trends in PAH in the case review and literature: 1) PAH decreases significantly after treatment, which was statistically significance (*p* < 0.05). The decrease in PAH after surgery was more significant than in the interventional closure group. 2) PAH decreased during follow-up in the interventional closure group, but was not statistically significant in the case review, whereas it was statistically significant in the literature (*p* < 0.05). 3) PAH increased during follow-up in all surgical groups (*p* < 0.05). According to the meta-analysis (Figure 4), closure and surgery are feasible to reduce PAH in patients with PAH-CHD (*p* < 0.10, *I* ^2^ > 50%). Pulmonary artery pressure control was poor in the postoperative group (*I* ^2^ < 50%), whereas it was good in the closed group (*p* < 0.10, *I* ^2^ > 50%).

#### Drug Factors

**FIGURE 5:**
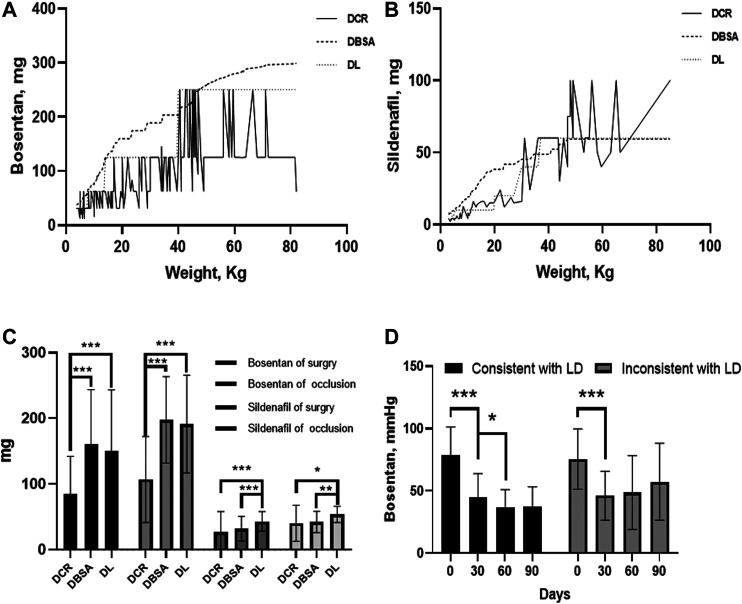
The comparision of the therapeutic and prognostic effects of drug factors. Three doses were including dose based on body surface area (DBSA), dose based on literature (DL), and based on case review (DCR). **(A)** Curves of 3 doses of bosentan with body weight. **(B)** Curves of 3 doses of sildenafil with body weight. **(C)** Dose comparison of 3 doses of bosentan and sildenafil administered in different operations. **(D)** The variation of pulmonary artery pressure concerning the consistency or inconsistency of bosentan literature dosage. *p < 0.05; **p < 0.01; ***p < 0.001.

In order to study the appropriate dose, we selected seven literature through criterion 2 (Table 4) (Mohamed and Ismail, 2012; Steinhorn et al., 2016; Omar et al., 2016; Hernando and Amed, 2006; Sinan et al., 2011; Robyn et al., 2011; Carmine et al., 2017). According to the literature, we listed the corresponding body surface area with different body weights and the calculated dose of two drugs based on the body surface area (Li, 2010). As shown in Figure 5, the actual doses of bosentan and sildenafil were insufficient when compared with the dosage in the literature and surface area calculated. The dosage in consistent with the literature and the dosage in inconsistent with the literature were divided into two groups. It was found that the literature group still had a statistically significant reduction in PAH after 30 to 60 days of follow-up (*p* < 0.05). On the other hand, the group that did not follow the literature dosage showed a sustained increase in PAH after 30 days of follow-up.

**TABLE 4:** Body surface area dosage and dosage in literature of bosentan and sildenafil.

	Weight, kg	Study	＜5	∼20	∼30	∼35	∼40	∼45	∼50	∼60	∼70
	Body surface area, m^2^	Li2010	0.035 × Weight + 0.1			1.2	1.3	1.4	1.5	1.6	1.7
Bosentan	Body surface area dosage, mg/day		(0.035 × Weight + 0.1) × 147			176	191	206	221	235	250
Sildenafil	Body surface area dosage, mg/day		(0.035 × Weight + 0.1) × 35			42	46	49	53	56	60
Bosentan	Dosage in literature, mg/day	Mohamed2011	2	NA	NA	NA	NA	NA	NA	NA	NA
		Steinhorn2016	4	NA	NA	NA	NA	NA	NA	NA	NA
		Instruction	NA	63	125	125	250	250	250	250	250
		MAX	4	62.5	125	125	250	250	250	250	250
Sildenafil	Dosage in literature, mg/day	Al Omar2016	8	NA	NA	NA	NA	NA	NA	NA	NA
		uslu2010	2	NA	NA	NA	NA	NA	NA	NA	NA
		baquero2006	4	NA	NA	NA	NA	NA	NA	NA	NA
		Robyn2011	NA	10	20	20	20	40	40	40	40
		Carmine2017	NA	NA	NA	NA	NA	NA	60	60	60
		MAX	8	10	20	40	60	60	60	60	60

NA means there is no relevant value in the references.

**FIGURE 6:**
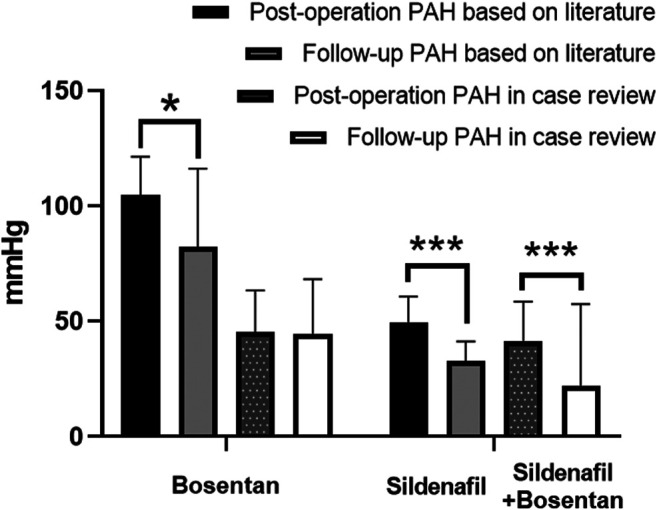
Comparison of postoperative pulmonary pressure reduction effects between bosentan and sildenafil in case review and literature. *p < 0.05; **p < 0.01; ***p < 0.001.

In order to study the postoperative treatment effect of these two drugs, two literature were screened out by criterion 3. Since no literature was found on postoperative combination therapy (sildenafil and bosentan), this study focused only on the effect of postoperative sildenafil monotherapy (Cheng et al., 2014; Yang, 2014). We found a statistically significant reduction in PAH in both the literature group of sildenafil and the case review group with combination therapy (*p* < 0.05). Bosentan reduced PAH only in the literature group, which was statistical difference in Figure 6 (*p* < 0.05).

#### Age Factors

We divided 235 patients into five groups (48 infants, 46 toddlers, 47 children, 22 adolescents, and 72 adults) (Table 5). The number of minors hospitalized for PAH-CHD was twice as many as adults (163:72). The best treatment outcome (36.14 mmHg) and the lowest recurrence rate (0.15%) were observed mainly in infants.

**TABLE 5:** Comparison of recurrence and therapeutic effect in 5 age groups (Infant, toddlers, children, adolescent, and adult).

	Age	Recurrence, %	Preoperative PAH, mmHg			Therapeutic effect, mmHg
			Mean	SD	N	
Infant	<1	0.15	67.33	17.71	48	36.14
Toddlers	1–6	0.28	68.46	21.80	46	28.75
Children	7–14	0.38	71.27	26.94	47	31.69
Adolescent	15–17	0.50	82.16	30.49	22	32.47
Adult	≥18	0.53	77.91	26.91	72	30.86

## Discussion

### Orthogonal Projections to Latent Structures-Discriminant Analysis and S-Plot

OPLS-DA could be applicable to the analysis from routine clinical examination data, and S-plot could be helpful to screen out the indicators. We believe that simple methods such as OPLS-DA and S-plot are extremely important for screening prognostic indicators, in terms of saving doctors’ time to read the checklist and saving patients’ medical examination costs than PCA. Although four indicators (PAH, RBC, HGB, and CO_2_) were screened out in this paper, few studies have used RBC, HGB, and CO_2_ as prognostic indicators.

### Cause Analysis of Elevated Pulmonary Arterial Hypertension

#### Reasons for the Operation

Combined with the literature and the case review in this study, surgery is inferior to closure. This phenomenon is widespread. The reason for this phenomenon might due to surgery requiring open chest and extracorporeal blood circulation. In contrast, the closure is less invasive and reduces the risk of infection. In this study, although the inclusion criterion for three times literature retrievals were consulted English and Chinese, almost most of the included literature was in Chinese (Suzuki et al., 2017; Akagi et al., 2018; Zwijnenburg et al., 2018). This might be because Chinese researchers might be more concerned with the follow-up of postoperative PAH, while researchers in other countries might pay more attention to the outcome of the operation itself. To improve patient survival, we believe that the postoperative follow-up of patients with PAH-CHD (detection of adverse factors and timely intervention) is very important. We hope that more researchers studying PAH will focus on the long-term postoperative follow-up to optimize the treatment and even provide a basis for treatment recommendations for patients with PAH-CHD.

#### Reasons for the Drug

The effect of combination therapy (bosentan and sildenafil) was more effective than that of bosentan. This result is consistent with the results of meta-analysis (Li et al., 2020). We propose that the main reason for poor postoperative control might be caused by insufficient dose and inappropriate medication. Most of the patients with PAH-CHD in our hospital were pediatric patients. Unfortunately, The drug dosage of these patients was not indicated on the packaging of bosentan and sildenafil. The commonly used method to determine the dosage of pediatric medicine is to convert it according to body weight, body surface area, or adult dosage. One study argued that doses should be calculated according to the 2/3 power of body weight (West, 2018). For example, Bosentan 60 kg adult dose is 250 mg/day, then the daily dose for a 10 kg toddler is 250/(60/10)^2/3^ ≈ 75 mg. However, no matter which method is used to calculate, the dosage of drugs in some patients was far from adequate. We also consider the efficacy of controlling postoperative pulmonary arterial pressure by standardizing patient dosing, which requires further study.

#### Reasons for the Age

The younger the patient’s age, the more effective the reduction in PAH. As the patient ages, the CHD might lead to cardiac remodeling and pulmonary artery function. Also, Also, damage to multiple organs may occur in the body due to chronic hypoxia, and a sudden increase in oxygen saturation does not reverse the damage. Therefore, we strongly recommend early operation (closure or surgery) for patients with CHD to improve the recovery outcome.

## Conclusion

For the first time, OPLS-DA combined with S-plot was used to analyze clinical examination data, and four indicators (PAH, RBC, HGB, and CO_2_) reflecting patients’ prognosis were found. We suggest that occlision therapy would be preferred in patients with PAH-CHD. To prevent recurrence, the dose should be formally prescribed according to the instructions or literature for use. The effect of combination therapy (bosentan and sildenafil) is more effective than that of bosentan. The younger the patient is, the more effective the treatment is.

## Data Availability

The original contributions presented in the study are included in the article/Supplementary Material, further inquiries can be directed to the corresponding author.
